# Effects of Different Mulching Practices on Soil Environment and Fruit Quality in Peach Orchards

**DOI:** 10.3390/plants13060827

**Published:** 2024-03-13

**Authors:** Lei Guo, Siyu Liu, Peizhi Zhang, Abdul Hakeem, Hongfeng Song, Mingliang Yu, Falin Wang

**Affiliations:** 1 College of Horticulture, Gansu Agricultural University, Lanzhou 730070, China; jaasgl@163.com; 2 Fruit Crop Genetic Improvement and Seedling Propagation Engineering Research Center of Jiangsu Province, Nanjing 210095, China; 3 Institute of Pomology, Jiangsu Academy of Agricultural Sciences, Jiangsu Key Laboratory for Horticultural Crop Genetic Improvement, Nanjing 210014, Chinamly@jaas.ac.cn (M.Y.)

**Keywords:** peach orchards, living grass mulch, soil nutrients, microbial community, fruit quality

## Abstract

Mulching practices have been used to improve peach growth and production across the globe. However, the impact of mulching on the physiochemical properties and soil characteristics of orchards remains largely unknown. This study aimed to decipher the impacts of various mulching patterns on the soil environment and the quality of *Prunus persica* fruit in “Zijinhuangcui”. Three treatments were set up, which included black ground fabric mulch (BF) and two living grass mulch treatments (HV: hairy vetch and RG: ryegrass). The results showed that different mulching treatments have different effects on soil, plant growth, and fruit quality. Living grass mulch treatments, especially the HV treatment, significantly improved soil nutrients by enhancing nitrogen-related indicators. Of note, the BF treatment had higher total phosphorus and available phosphorus contents than the HV and RG treatments. The HV treatment had the highest relative abundance of Proteobacteria (33.49%), which is associated with symbiotic nitrogen fixation, followed by RG (25.62%), and BF (22.38%) at the young fruit stage. Similarly, the abundance of *Terrimonas*, which has a unique nitrogen fixation system at the genus level, was significantly higher in the living grass mulch (HV, 1.30–3.13% and RG, 2.27–4.24%) than in the BF treatment. Living grass mulch also promoted tree growth, increased fruit sugar content, sugar-related components, and sugar-acid ratio, and reduced the acid content. Collectively, the findings of this study show that living grass mulch can promote tree growth and improve fruit quality by improving soil fertility, bacterial diversity, and richness.

## 1. Introduction

Peaches (*Prunus persica* [L.] Batsch) are the world’s second most important deciduous fruit crop in terms of their economic value. China is the world’s leading producer of peaches, with an annual output value of 15.02 million tons [1]. However, China faces numerous problems in the cultivation and management models of peaches compared with other countries. Fruit farmers follow traditional farming concepts and believe that weeds are detrimental to orchards. Farmers implement clear tillage management of orchard land, leading to reduced soil fertility, deterioration of the orchard ecological environment, and direct and indirect reduction in fruit quality and yield, which is not conducive to the development of the fruit industry [2].

Orchard soil management methods mainly include full tillage and mulching [3]. Full tillage involves the removal of weeds to prevent them from competing with trees for nutrients. In the short term, full tillage also prevents soil compaction, suppresses pests and diseases, and facilitates organic matter mineralization, making the soil more conducive for fruit trees to absorb nutrients [4]. Mulching practices include the use of widely planted living grasses and black ground fabric mulches in orchards. Both mulching practices effectively prevent soil erosion, improve soil quality [5,6], and control weeds [7]. For instance, intercrop ryegrass (*Lolium perenne* L.) cover and inter-row cornstalk mulch promote soil organic matter accumulation [8]. However, different covering materials have different effects. For example, the application of organic mulching measures (intercrop ryegrass cover and inter-row cornstalk mulch) is more beneficial to improving soil quality than inorganic mulching measures (black ground fabric mulch) in a semi-arid apple orchard despite both mulching measures increasing the soil moisture content [8]. Studies also postulate that organic mulching practices (ryegrass intercropping cover and cornstalk mulch) increase the diversity of soil immune functions associated with carbon and nitrogen cycling, while inorganic mulching (black ground fabric mulch) practices reduce this diversity [9].

Living grass mulching is an important practice for controlling soil, water, and nutrient loss in orchards [10,11]. It involves selecting suitable grass species for artificial sowing in the entire orchard or between fruit tree rows or removing unsuitable grasses from the native weeds and retaining some for natural grass growth. Living grass mulching is an efficient soil management method and can improve soil fertility and microbial activity compared to full tillage [12,13,14,15,16,17]. Living grass mulching is now being increasingly used in orchards in China. It increases the soil nutrients, improves the soil structure, and reduces the degree of mineralization of soil organic matter in the orchard [18,19,20]. Monteiro and Lopes [21] postulated that natural grass is more conducive to achieving a balanced supply of mineral elements and improving fruit yield and quality. Moreover, numerous studies have shown that living grass mulch treatments in different regions positively improve the orchard environment. White clover (*Trifolium repens* L.), lavender (*Astragalus sinicus* L.), alfalfa (*Medicago sativa* L.), and other leguminous plants have been successfully used in apple and pear orchards to increase the available nitrogen content in the soil [22,23]. Neilsen et al. [24] also reported that growing grass in apple orchards increases the organic matter content. Similarly, Vicente-Vicente et al. [25] found that growing grass in olive orchards increased the soil’s organic matter content. Most studies have also reported that orchard grass significantly increases the soluble solids and sugar content of fruits [26] and reduces acidity [27,28]. Living grass mulch also increases single fruit weight and improves fruit appearance and quality. However, it can significantly reduce tree growth vigor and thickening of fruit trees [29,30].

The existing studies have mostly focused on the impact of orchard covering measures on the soil’s physical and chemical properties. Physical and chemical indicators can reflect changes in the dynamic life systems of soil, such as the cycle of biogeochemical substances, promotion of plant growth, decomposition of organic matter, and the various interactions of soil physics [31]. Furthermore, microorganisms are the core driving force for soil nutrient cycling and quality improvement. Soil fertility is closely associated with the types and quantities of soil microorganisms. Moreover, changes in soil properties caused by soil microorganisms on agricultural management measures, such as organic matter content and soil moisture availability, are very sensitive to plant growth. Different mulching measures can change the composition and diversity of microbial communities by improving the soil’s physical and chemical environment [32]. Living grass mulch increases the diversity of soil microorganisms, reduces the proliferation of soil pathogenic bacteria, and promotes the formation of new soil microbial communities [33,34]. For example, the roots of apples, citrus, and bayberry form a symbiotic relationship with microorganisms, which promotes the absorption and circulation of nutrients and improves the stress resistance of the tree [35]. Some studies postulate that the artificial living grass mulch also increases the number and activity of beneficial soil microorganisms in orchards, improves soil nutrients, and accelerates material circulation, thereby increasing fruit yield and quality [26].

Although studies have reported the effects of mulching measures on the soil microbial community structure, the response of microorganisms to soil environmental changes and the resulting impact on fruit quality remains unclear. This study employed long-term positioning experiments to decipher the characteristics of soil microbial community structure under different multi-year mulching measures, combined with soil environmental factors, to comprehensively evaluate the effect of mulching measures on orchard soil quality improvement and their impact on fruit quality. The findings of this study provide a theoretical basis and technical support for selecting and promoting soil management measures that benefit the region’s environmental and sustainable development of the peach industry.

## 2. Results

### 2.1. Effects of Different Mulching Practices on Soil Physiochemical Properties and Mineral Contents

**Figure 1:**
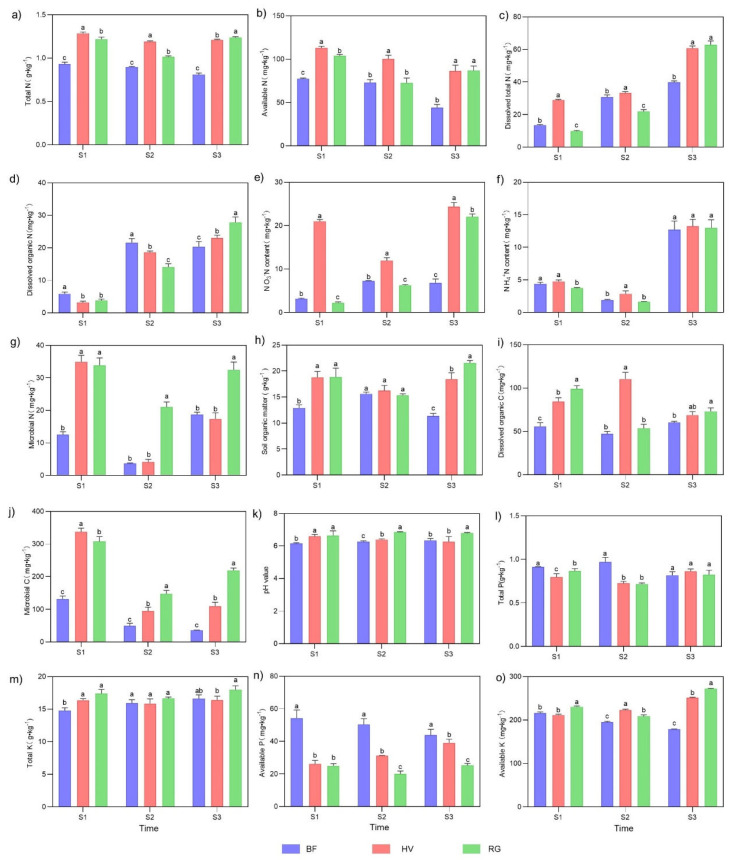
Effects of mulching practices on soil-related chemical indicators. (**a**) total nitrogen (TN); (**b**) available nitrogen (AN); (**c**) dissolved total nitrogen (DTN); (**d**) dissolved organic nitrogen (DON); (**e**) NO_3_^−^-N content; (**f**) NH_4_^+^-N content; (**g**) microbial nitrogen (MN); (**h**) soil organic matter (SOM); (**i**) dissolved organic carbon (DOC); (**j**) microbial carbon (MC); (**k**) pH value; (**l**) total phosphorus (TP); (**m**) total potassium (TK); (**n**) available potassium (AP); and (**o**) available potassium (AK); S1, the young fruit stage (30 April 2022); S2, the fruit mature stage (30 June 2022); S3, after the fruit harvest stage (30 September 2022); BF, black ground fabric mulch; HV, living hairy vetch mulch; RG, living ryegrass mulch. Boxes with different lowercase letters indicate significant differences between different mulch treatments based on the LSD test (*p* < 0.05).

The living grass mulch increased the soil nitrogen, potassium, and organic matter, while the black ground fabric mulch increased phosphorus in 2017–2022 (Table S1). This study further analyzed the soil changes in three different fruit growth stages in 2022. Notably, both the living grass mulch and black ground fabric mulch had an impact on the physical and chemical properties of the soil. The living grass mulch treatments were more conducive to the improvement of soil nutrients and physiochemical properties. Of note, the HV treatment had a significant effect on nitrogen-related indicators (TN, AN, DTN, NO_3_^−^-N, and NH_4_^+^-N) (Figure 1 and Table S2).

Though the total nitrogen (TN) and available nitrogen (AN) contents showed a decreasing trend during the fruit growth period, TN and AN content in the living grass mulch treatments (HV and RG) were higher than those in the BF treatment. TN and AN content in HV were significantly higher than those of RG, but the difference between the two was not significant after the harvest stage (S3) (Figure 1a,b). The dissolved organic nitrogen (DON) and dissolved total nitrogen (DTN) content generally showed an increasing trend. However, DON was mainly significantly higher in the BF at the young fruit stage (S1) and fruit mature stage (S2). Compared to the HV and RG treatments, DON in BF increased by 14.53–86.67% (HV) and 53.30–53.52% (RG) at the S2 and S1 stages, respectively (Figure 1c,d and Table S2). The contents of soluble total nitrogen (DTN), NO_3_^−^-N, and NH_4_^+^-N in the HV treatment were significantly higher than the other two treatments (BF and RG) in all stages. However, NO_3_^−^-N was significantly higher at the young fruit stage (6.62-fold and 9.30-fold) (S1) (Figure 1e,f and Table S2). Microbial nitrogen (MN) and microbial carbon (MC, except for the S1 stage) under the RG treatment were significantly higher than under the other two treatments (BF and HV) (Figure 1g,j). The organic matter content (SOM) at the S1 and S3 stages was significantly higher under the HV and RG treatments than under the BF treatment. Compared to the BF, the SOM content increased by 45.32% and 45.78% (S1) and 61.72% and 89.51% (S3) under the HV and RG treatments, respectively (Figure 1h). Compared to the BF treatment, the dissolved organic carbon (DOC) content in the HV and RG treatments increased by 14.15–133.93% and 13.53–78.04%, respectively, during the three stages (S1, S2, and S3). Notably, the HV treatment had the highest DOC content, reaching 110.46 mg·kg^−1^ at the S2 stage (Figure 1i and Table S2). The pH value fluctuation ranges under the BF, HV, and RG treatments were 6.15~6.34, 6.26~6.59, and 6.63~6.85, respectively, revealing a slight increase under the living grass mulch treatments (Figure 1k). The contents of total potassium (TK) and available potassium (AK) in soil under living grass mulch treatment (HV and RG) were higher than the corresponding contents under the BF treatment, with the contents being highest under the RG treatment in most periods. The content of available potassium (AK) in RG and HV increased significantly at the S3 stage compared to the BF treatment, with an increase of 52.16% and 40.68%, respectively (Figure 1m,o). However, the contents of available phosphorus (AP) in the BF treatment were significantly higher than those in living grass mulch treatment during the three stages (S1, S2 and S3). The total phosphorus (TP) content in the BF treatment was also significantly higher than in the living grass mulch treatment at theS1 and S2 stages (Figure 1l,n).

### 2.2. Effects of Different Mulching Practices on Soil Bacterial Communities

#### 2.2.1. Effect of Mulching Practices on Soil Bacterial Abundance and Composition

**Figure 2:**
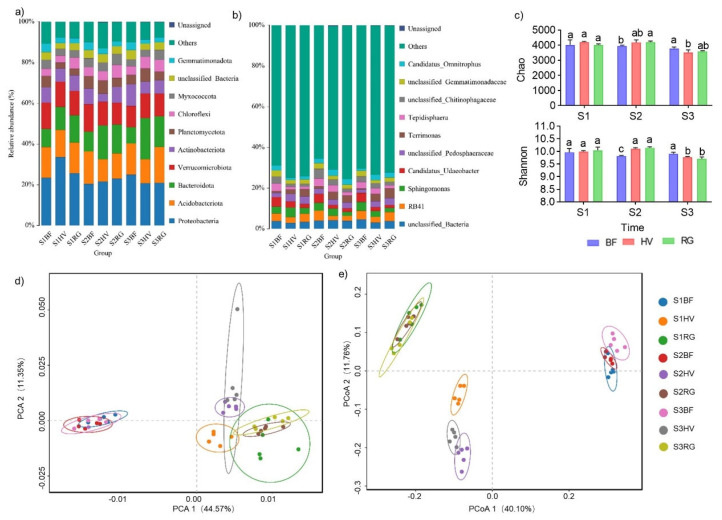
Response of relative abundance, alpha diversity index, principal component analysis (PCA), and principal coordinate analysis (PCoA). (**a**,**b**) represent the composition and distribution of relative abundance; (**c**) represents the Chao and Shannon diversity index; (**d**,**e**) PCA and PCoA analysis in the BF, HV, and RG treatments for the S1, S2, and S3 stages. S1, the young fruit stage (30 April 2022); S2, the fruit mature stage (30 June 2022); S3, after the fruit harvest stage (30 September 2022); BF, black ground fabric mulch; HV, living hairy vetch mulch; RG, living ryegrass mulch. Boxes with different lowercase letters indicate significant differences between different mulch treatments based on the LSD test (*p* < 0.05).

The relative abundance distribution of soil bacteria included 12 phyla but with different compositions in the BF, HV, and RG treatment groups (Figure 2a and Table S3). The top five phyla based on their relative abundance were Proteobacteria (20.40~33.49%), Acidobacteriota (10.95~17.73%), Bacteroidota (7.99~20.17%), Verrucomicrobiota (10.43~13.46%), and Actinobacteriota (3.89~10.67%) (Figure 2a). The relative abundance of Proteobacteria was significantly high under the HV treatment (33.49%) followed by the RG (25.62%) and BF (22.38%) treatments in S1 stage. However, there was a significant decrease in Proteobacteria abundance under the BF treatment in the S2 stage (Figure 2a). The relative abundance of Acidobacteriota in the RG treatment exhibited a decreasing trend and then increased, while the corresponding abundance in the BF treatment had minimal changes across the three stages (15.07–15.95%). The relative abundance of Acidobacteriota in the HV treatment was the lowest at each stage. Compared to BF and RG, Acidobacteriota abundance decreased by 1.69~5.01% and 4.4~7.02%, respectively (Table S3). Similarly, the relative abundance of Actinobacteriota significantly decreased under the HV treatment but increased significantly under the BF treatment (0.93~4.48%). The relative abundance of Bacteroidota significantly increased under the HV treatment (2.38~12.18% and 4.40~7.02%) at the S2 and S3 stages compared to under the BF and RG treatments. The relative abundance of Verrucomicrobiota decreased in the HV and RG treatments (1.77% and 2.83%) in the S2 stage but increased (1.65%, and 0.66%) significantly in the S3 stage compared to the BF treatment (Table S3).

The relative abundance distribution included up to 12 bacterial genera but with different compositions in the BF, HV, and RG treatment groups (Figure 2b and Table S4). The top five bacterial genera based on the relative abundance were *RB41*, *Sphingomonas*, *Candidatus_Udaeobacter*, *Terrimonas*, and *Tepidisphaera* (Figure 2b and Table S4). The relative abundance of *RB41* was lowest under the HV treatment. The relative abundance of *Sphingomonas* under the HV treatment was significantly higher than that of the RG and BF treatments at the S1 stage. However, *Sphingomonas* abundance under the BF treatment was significantly higher than that of living grass mulch treatment (HV and RG) at the S3 stage. In the same line, the relative abundances of *Candidatus_Udaeobacter* and *Tepidisphaera* significantly increased under the BF treatment at the S1, S2, and S3 stages compared to under the HV and RG treatments. In contrast, the relative abundance of *Terrimonas* significantly decreased under the BF treatments at the S1, S2, and S3 stages compared to under the HV and RG treatments (Table S4).

#### 2.2.2. Diversity Analysis of Bacterial Communities

The diversity and richness of soil bacteria increased significantly under living grass mulch at the S2 stage. However, the increase was not significant at the S1 stage and decreased at the S3 stage (Figure 2c). The Shannon and Chao index of bacteria were measured to evaluate the alpha diversity of the soil microbial community under different mulching practices. The Shannon index is proportional to the diversity of microorganisms, while the Chao index is proportional to the abundance of microorganisms. Higher values of these indexes denote a greater diversity and abundance of the microbial community. The Shannon and Chao indexes were similar among the treatments at the S1 stage. However, the Shannon and Chao indexes of the HV and RG treatments at the S2 stage (Shannon indexes were 10.11 and 10.14, respectively, and Chao indexes were 4178.66 and 4196.29, respectively) were significantly higher than those of the BF treatment (9.81 and 3960.19). The diversity index of the BF treatment was significantly higher than that of the HV and RG treatments for S3 stages (Figure 2c and Table S5).

Beta diversity is an index that measures the similarity of bacterial communities between different samples. The principal component analysis (PCA) and principal coordinate analysis (PCoA), used to identify the soil bacterial community structure in BF, HV, and RG-treated samples, revealed significant differences between the treatments (Figure 2d,e). PCA1 and PCA2 explained 44.57% and 11.35%, respectively (Figure 2d), while PCoA1 and PCoA2 explained 40.10% and 11.76%, respectively (Figure 2e). PCA and PCoA analysis results showed a separating in the living grass mulch and BF treatment. There was an intersection between the living grass mulch despite the HV and RG treatments having many common OTUs (Figure S1). This finding suggested that different treatments had their own special microbial community structures, and the bacterial community structures under the living grass mulch had certain similarities.

### 2.3. Effects of Mulching Practices on Tree Growth Indicators and Fruit Qualities

#### 2.3.1. Effects of Mulching Practices on Tree Growth and Leaf Mineral Element Content

**Figure 3:**
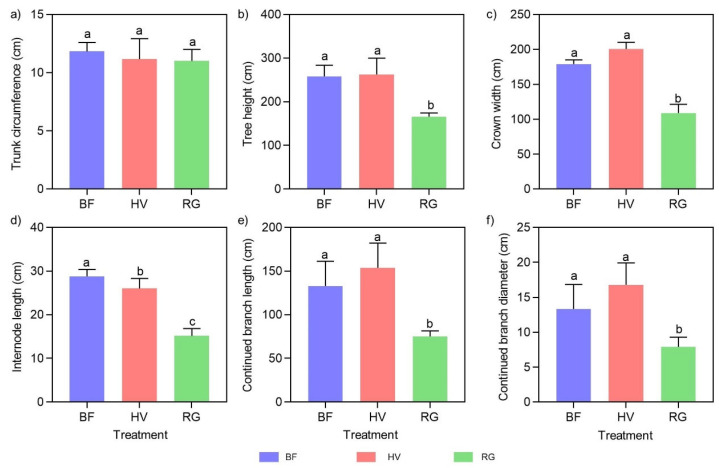
Growth index of peach tree under different treatments in 2022. (**a**) trunk circumference (cm); (**b**) tree height (cm); (**c**) crown width (cm); (**d**) internode length (cm); (**e**) continued branch length (cm); (**f**) continued branch diameter (cm). BF, black ground fabric mulch; HV, living hairy vetch mulch; RG, living ryegrass mulch. Boxes with different lowercase letters indicate significant differences between different mulch treatments based on the LSD test (*p* < 0.05).

The tree growth under the HV and BF treatments was better than in the RG treatment. There was no significant difference in tree height, crown width, continued branch length, and thickness between the HV and BF treatments, but a significant difference in the same parameters was observed between the HV or BF and RG treatments. Therefore, among the two living grass mulching treatments, the HV treatment had a superior promotional effect on the growth of peach trees than the RG treatment (Figure 3 and Table S6).

The total nitrogen (TN) content of leaves under the HV treatment exhibited a decreasing trend and then increased, while the total phosphorus (TP) and total potassium (TK) contents showed an opposite trend at different stages. The TN and TP of leaves under the RG treatment showed a gradual increase, while the TK content decreased and then increased. HV significantly promoted the accumulation of TN at the S1 stage compared to BF but significantly reduced the TK content. RG significantly promoted an increase in the TK content but significantly reduced the TN and TP contents in the leaves. HV significantly promoted the accumulation of TN, TK, and TP, while RG significantly promoted the increase in TK and TP contents at the S2 stage compared to BF. The TK and TP of RG and HV increased by 38.42% and 41.70%, and 41.06% and 43.91%, respectively (Figure S2 and Table 1). Except for the significant increase in the TP content under the RG treatment compared to the BF treatment, there was basically no significant difference in the mineral element of leaves under each treatment at the S3 stage (Figure S2).

#### 2.3.2. Effect of Mulching Practices on Peach Fruit Quality

**Figure 4:**
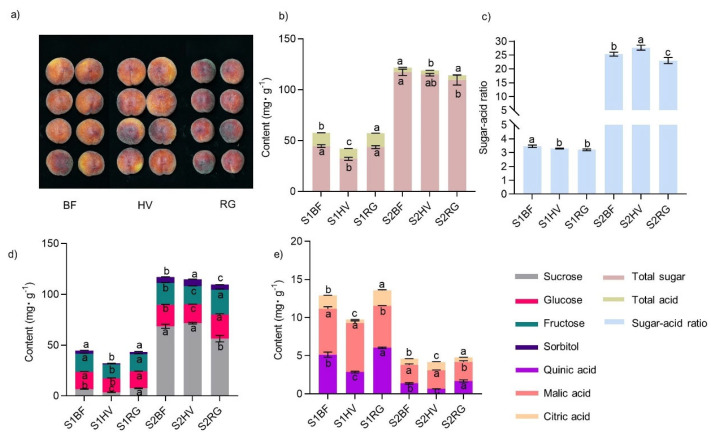
Fruit quality of peach under different mulch treatments. (**a**) pictures of fruit mature stages under different treatments; (**b**) total sugar and total acid content of fruits at the young fruit and mature stages under different treatments; (**c**) sugar-acid ratio of fruits at the young fruit and mature stages under different treatments; (**d**) content of each sugar component groups at the young fruit and mature stages under different treatments; (**e**) content of each acid component at young fruit and mature stages under different treatments. S1, the young fruit stage (30 April 2022); S2, the fruit mature stage (30 June 2022); BF, black ground fabric mulch; HV, living hairy vetch mulch; RG, living ryegrass mulch. Boxes with different lowercase letters indicate significant differences between different mulch treatments at the same stage based on the LSD test (*p* < 0.05).

The fruit phenotypes revealed significant differences in the quality of peach fruits between the BF and the living grass mulch treatments (RG and HV). Of note, the single fruit weight, length, width, and side diameter showed a trend of HV > BF >RG (Figure 4a and Table 2). The a* value of fruits under the RG treatment was significantly higher than that of fruits in the other treatments. In contrast, the h* value of fruits under the RG treatment was significantly lower than that of fruits in the other treatments. These findings suggested that the RG and BF treatments promoted the coloring of peaches, with RG having a better effect, while the HV treatment had a less effective coloring effect on the peach fruits (Table 2).

The total sugar, total acid content, sugar-acid ratio, and changes in each sugar and acid component of peaches are important indicators for measuring whether the intrinsic quality of the fruit is excellent. Herein, there were significant differences in fruit quality under each treatment. Peaches under the HV treatment had the best intrinsic quality. HV treatment increased the total sugar content and sugar components of the fruit but reduced the total acid content, which was manifested by a higher sugar-acid ratio (Figure 4b–d and Table S7). The contents of sucrose, glucose, fructose, sorbitol, total sugar, quinic acid, citric acid, and total acid under the HV treatment were significantly lower than those under the RG and BF treatments at the S1 stage. The overall effect of RG on the accumulation of fruit sugar was not significant, but the contents of quinic acid, citric acid, and total acid were higher. At the S2 stage, the contents of glucose, fructose, quinic acid, and total acid were still significantly lower than those of the other treatments. However, the contents of sucrose, sorbitol, and citric acid had increased, and the sugar-acid ratio was at its highest. The contents of glucose, fructose, and quinic acid under the RG treatment were the highest and significantly higher than those of the HV and BF treatments. In the same line, the sucrose, sorbitol, and sugar-acid ratios were significantly lower under the RG treatment compared to those of the other two treatments (Figure 4b–d and Table S7). BF and HV treatment increased the total sugar content and sugar components of the fruit and reduced the total acid content, which was manifested in a higher sugar-acid ratio (Figure 4b–d and Table S7).

### 2.4. Correlation Analysis between Soil Physicochemical Factors and Soil Microbial Communities

**Figure 5:**
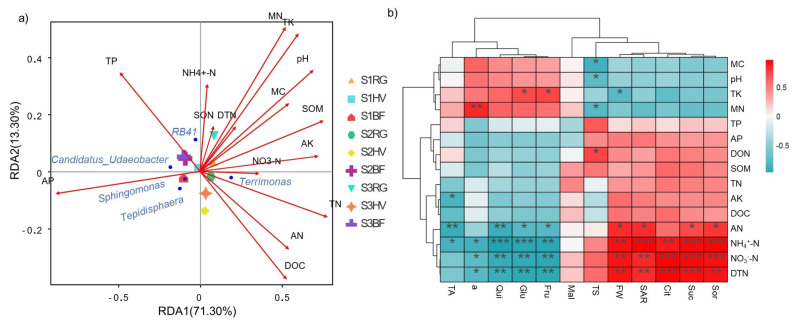
Correlation analysis of environmental factors, fruit quality, and microbial flora under different treatments. (**a**) RDA analysis of environmental factors and microbial flora; (**b**) correlation analysis of fruit quality and soil physical and chemical factors. S1, the young fruit stage (30 April 2022); S2, the fruit mature stage (30 June 2022); S3, after the fruit harvest stage (30 September 2022); BF, black ground fabric mulch; HV, living hairy vetch mulch; RG, living ryegrass mulch. TN, total nitrogen; AN, available nitrogen; DTN, dissolved total nitrogen; DON, dissolved organic nitrogen; MN, microbial nitrogen; SOM, soil organic matter; DOC, dissolved organic carbon; MC, microbial carbon; TP, total phosphorus; TK, total potassium; AP, available potassium; AK, available potassium; SON, soluble organic nitrogen; Sor, Sorbitol; Suc, sucrose; Cit, citric acid; SAR, sugar-acid ratio; FW, fruit weight; TS, total sugar; Mal, malic acid; Fru, fructose; Glu, glucose; Qui, quinic acid; TA, total acid. * represents signiffcance *p* < 0.05, ** represents signiffcance *p* < 0.01, *** represents significance *p* < 0.001.

Environmental factors and microbial flora interact with each other. A redundancy analysis (RDA) was thus used to evaluate the relationship between environmental factors and microbial flora in different treatments at the genus level. The explanation rates of the first and second ordering axes of RDA were 71.30% and 13.30%, respectively, and cumulatively explained 84.6% of the microbial changes (Figure 5a). There were significant correlations between most physical and chemical factors, especially in the areas associated with nitrogen nutrients, under the living grass mulch treatments at different periods. The bacterial genus *Terrimonas* had a significant positive correlation with TN, AN, SOM, DOC, and MC. The genus *Sphingomonas* had a significant positive correlation with AP but a significant negative correlation with TK, AK, SOM, and pH. Moreover, there were significant negative correlations between *Candidatus_Udaeobacter* and *Tepidisphaera* and SOM, MC, and MN. *RB41* and DOC had a significant negative correlation (Figure 5a).

### 2.5. Correlation Analysis between Soil Physical and Chemical Properties and Fruit Quality

Figure 5b shows a correlation analysis between indicators associated with peach fruit quality at maturity and the physical and chemical properties of the peach orchard soil at 0~20 cm. Soil available nitrogen (AN), NO_3_^−^-N, NH_4_^+^-N, dissolved total nitrogen (DTN), and other indicators were closely associated with fruit quality indicators. Glucose (Glu) and fructose (Fru) contents were significantly positively correlated with soil total potassium (TK) content but significantly negatively correlated with soil AN, NO_3_^−^-N, NH_4_^+^-N, and DTN. Fruit sucrose (Suc) and sorbitol (Sor) had significant positive correlations with soil AN, NO_3_^−^-N, NH_4_^+^-N, and DTN. Total sugar (TS) was positively correlated with dissolved organic nitrogen (DON) but significantly negatively correlated with microbial carbon (MC), microbial nitrogen (MN), and pH. The quinic acid (Qui) content of the fruit had a significant negative correlation with AN, NO_3_^−^-N, NH_4_^+^-N, and DTN. In contrast, the citric acid (Cit) content had a significant positive correlation with NO_3_^−^-N, NH_4_^+^-N, and DTN. There was a significant negative correlation between total fruit acid and available potassium (AK), AN, and NH_4_^+^-N.

In terms of fruit color, a* (the degree of redness of the peel) was positively correlated with the MN content but had a significant negative correlation with NO_3_^-^-N, NH_4_^+^-N, and DTN. The fruit weight (FW) had a significant positive correlation with AN, NO_3_^-^-N, NH_4_^+^-N, and DTN. Single fruit weight (FW) and sugar-acid ratio (SAR) were significantly positively correlated with DTN, AN, NO_3_^−^-N, and NH_4_^+^-N but were significantly negatively correlated with microorganisms. Total acid (TA) had a significant negative correlation with AN, AK, and NH_4_^+^-N. Total sugar (TS) was significantly positively correlated with (DON) and soluble organic nitrogen (SON) but significantly negatively correlated with MN, MC, and pH. Notably, the correlation between SAR, DTN, AN, NO_3_^−^-N, NH_4_^+^-N, dissolved organic carbon (DOC), TA, FW, and sugar in the peaches reached a significant or extremely significant level. Similarly, the correlation between SON, MN, MC, pH, and TS in the peaches reached a significant or extremely significant level. These results showed that nitrogen accounted for the largest proportion of correlation, indicating that soil nitrogen can significantly affect the quality of peach.

## 3. Discussion

### 3.1. Effects of Mulching Practices on Soil Characteristics, Microbial Community, and Nutrients

Mulching practices are core factors in pedology because of their effects on the soil organic matter, soil fertility, aggregate stability, soil productivity, biological activities, plant nutrient cycling, fruit quality, and pomological research [36]. Biological, chemical, and physical processes in soil often require several years to display noticeable changes. Previous studies on mulching treatments have consistently involved long-term experiments. Examples include plastic mulch and nitrogen fertilization in semi-arid farmland (9 years, 2009–2018) [37], gravel mulching and straw mulching in the Loess Plateau of China (10 years, 2008–2018) [38], no-tillage with varying straw mulching frequencies (10 years, 2007–2017) [39], and living grass mulch in pear orchards with natural grass planting for 1, 2, 3, 4, and 5 years; southern citrus orchards from 2001 to 2015; wolfberry orchards from 2018 to 2020) [36,40,41]. Notably, living grass mulch had a more positive impact on soil organic matter in southern citrus orchards [40], pear orchards [36], and wolfberry orchards [41], leading to increases of 1.45%, 12.59%, and 37%, respectively. Our study, spanning several years (2016–2022), has further confirmed that the effects of mulching treatments on soil require an extended period to manifest (Table S1). Through this approach, we explored the correlation between soil characteristics and the quality of peach fruits at different stages. Similarly, HV and RG treatments significantly increased the soil organic matter at the S1 and S3 stages by 45.32% and 45.78%, and 61.72% and 89.51%, respectively. This phenomenon is attributed to the fact that the litter produced under the living grass mulch enters the soil and is continuously decomposed and transformed in the soil. Additionally, nitrogen is an important factor in the growth and developmental process of soil physiochemical properties because it enhances the ability of microbial diversity to utilize carbon sources, thereby promoting the increase in microbial carbon biomass [42]. In this study, the contents of TN, AN, MN, DOC, and MC in the two living grass mulch treatments (HV and RG) were significantly increased compared to the corresponding contents under the BF treatment in the three stages. Of note, the HV treatment had a more significant impact on nitrogen-related indicators (TN, AN, DTN, NO_3_^−^-N, NH_4_^+^-N). Similarly, Tang et al. [43] reported that white clover mulch significantly increased the soil TN content in apple orchards, while ryegrass had no significant effect. This phenomenon is attributed to the fact that both hairy vetch and white clover are leguminous crops and have strong nitrogen fixation capabilities [44]. In addition, the DON of BF was the highest in S1 and S2 but the lowest in S3, which is attributed to the decay of grass. The sources of DON mainly include litter, soil humus, intermediate products of organic matter decomposition, applied organic fertilizers, soil microbial biomass and residues, crop straw, metabolites and secretions of microorganisms and roots, and rainfall leaching, among other sources [45,46,47]. In this study, the two grasses were still in the vigorous growth stage during the S1 period, and thus, the grass may have absorbed DON from the soil, thereby reducing its content. In the S2 period, the grass had just died and had not started to decompose, causing the DON content to be generally lower than in BF. In the S3 period, the content of DON increased because the grass had started decomposing.

Soil nutrients are directly proportional to the functions of soil microbial activities. Of note, soil nutrients and soil microbes play a pivotal role in nutrient cycling and absorption [48], especially the cycle of carbon and nitrogen, including sedimentation, ammonification, nitrogen fixation, nitrogen fixation, ammonia oxidation, nitrification, and denitrification [49,50,51]. Previous studies postulate that the ecological diversity of nitrogen-fixing bacteria are generally photoautotrophic bacteria and include Proteobacteria, Agrobacterium, Green Sulfur Bacteria, Cyanobacteria, and Firmicutes [52]. Herein, the relative abundance of Proteobacteria, which was the dominant bacterial phylum, was significantly higher at the young fruit stage under the HV treatment (33.49%) than under the RG treatment (25.62%) and BF treatment (22.38%) (Figure 2a and Table S4). The abundance of Proteobacteria was hypothesized to be beneficial for enhancing soil nitrogen nutrients in the peach orchards. Similarly, the abundance of *Terrimonas* plays a significant role in nitrogen fixation, thus enhancing the utilization of atmospheric nitrogen for the growth and development of plants [53]. In this study, the genus *Terrimonas* was positively associated with TN, AN, SOM, DOC, and MC in the different mulch treatments. The significant positive correlation was beneficial to the accumulation of C and N nutrients. *Candidatus_Udaeobacter* is a functional bacterium responsible for secreting antibiotics in soil, which potentially remove trace gases [54]. It colonizes the soil nutrients and can survive by obtaining amino acids and vitamins from the soil [55]. Gao et al. [56] reported that the abundance of *Candidatus_Udaeobacter* is positively correlated with the concentrations of NH_4_^+^-N, NO_3_^−^-N, and TN, and it is thus believed that it is associated with nitrogen conversion. However, in this study, *Candidatus_Udaeobacter* had a significant negative correlation with TN, AN, MN, and NO_3_^−^-N, which was potentially associated with the different soil quality and nutrient conditions. Studies postulate that *Sphingomonas* are associated with phosphorus solubilization [57]. In this study, *Sphingomonas* was significantly positively correlated with AP but significantly negatively correlated with TK, AK, SOM, and pH. These correlations showed that the abundance of *Sphingomonas* was higher in the BF treatment at the mature fruit stage and after the fruit harvest stage. Moreover, the AP content under the BF treatment was significantly higher than that under the living grass mulch treatment.

### 3.2. Effects of Mulching Practices on Tree Growth and Fruit Quality

Different mulching treatments cause changes in the soil’s physical and chemical properties and microorganisms in orchards. Living grass mulching improves the physical and chemical properties of the soil and increases beneficial microorganisms. These changes improve the small microenvironment of the orchard, thereby promoting the growth of fruit trees and increasing fruit quality and yield [58]. In this study, living grass mulch improved soil fertility, thereby promoting the growth of peach trees. The HV treatment promoted tree height, crown width, continued branch length, and thickness compared to the BF treatment. However, the RG treatment significantly reduced these indexes. This phenomenon was attributed to ryegrass, which belongs to the family Gramineae and thus absorbs nutrients from the soil during its growth, forming a competitive relationship with fruit trees, thereby affecting growth. The hairy vetch is leguminous, and the rhizobia in its roots can absorb nitrogen from the air, thereby increasing the tree’s nitrogen absorption, which promotes the growth of fruit trees. The content of mineral elements in leaves can effectively reflect the nutritional level of fruit trees [59]. In this study, the leaf nitrogen content increased significantly under the HV treatment. In contrast, TerAvest et al. [60] reported that living grass mulch significantly weakened the vegetative growth of apple trees, possibly because of the competition with the grass for soil water, which caused mild water stress in the fruit tree, inhibiting the vegetative growth [21,61].

Herein, the two types of living grass mulch had opposite effects on the peach fruits. HV increased the fruit size, but the coloring was slightly worse, while fruits under the RG treatment were smaller but had good coloring. These differences were attributed to variations in the soil nitrogen content. Studies postulate that low nitrogen levels in plants promote the accumulation of flavonoids [62]. Many studies have also reported that nitrogen supply has a negative correlation with fruit color [63,64]. Similarly, there was a negative correlation between fruit color and nitrogen-related components in this study. The fruit color under the BF and RG treatments, which had a lower nitrogen content, was darker than that of the HV treatment, which had a higher nitrogen content. The HV treatment significantly increased the contents of sucrose, sorbitol, and citric acid, and significantly reduced the total acid content which substantially increased the sugar-acid ratio than in the other two treatments (BF and RG). This finding was similar to that of Monteiro and Lopes et al. [21], who reported that grassland treatment did not affect grape yield or fructose accumulation in the fruit but reduced grape acidity and increased total phenolics and anthocyanin compared to the control (land tillage). In this study, sucrose and sorbitol in fruits were the most relevant indicators for taste, while malic acid and citric acid were the main contributors to optimal acidity [65]. Fruits under the HV treatment had high sucrose and sorbitol contents and a high sugar-acid ratio, indicating that the HV treatment significantly improved fruit quality. Moreover, there were significant positive correlations between sucrose, sorbitol, citric acid, and sugar-acid ratio and DTN, NO_3_^−^-N, and NH_4_^+^-N under the HV treatment. In the same line, Rubio Ames et al. [66] postulated that the nitrogen content is significantly positively correlated with single fruit weight, which is consistent with the findings of this study.

## 4. Materials and Methods

### 4.1. Overview of the Test Sites

The experiment was conducted between September 2016 and October 2022 in the peach orchard of the Fruit Research Institute of the Jiangsu Academy of Agricultural Sciences, Nanjing, Jiangsu province, China. The experimental areas lay 118°87′ E, 32°03′ N, with an altitude of 11 m, annual precipitation of 1000~1100 mm, an average annual temperature of 15.7 °C, an annual accumulated temperature of about 4800 °C (with 0 °C as the base temperature), an average sunshine time of 1900 h and a frost-free period of 220~240 d throughout the year. The basic chemical properties of the soil before the test were pH 6.88, soil organic matter content 13.87 g·kg^−1^, total nitrogen 0.89 g·kg^−1^, total phosphorus 0.85 g·kg^−1^, total potassium 10.23 g·kg^−1^, and available nitrogen 55.56 mg·kg^−1^, available phosphorus 16.12 mg·kg^−1^, and available potassium 132.77 mg·kg^−1^. No chemical fertilizers were used in the peach orchard during the experimental period. 

An organic fertilizer (rapeseed cake) was applied in the holes before rotary tillage in early October every year at a rate of 2 kg/tree (organic matter, nitrate nitrogen, total nitrogen, total phosphorus, and total potassium: 204 g·kg^−1^, 2.88 g·kg^−1^, 58.77 g·kg^−1^, 3.31 g·kg^−1^, and 6.62 g·kg^−1^). The application site was along the row of the peach tree, 30 cm away from the main trunk. The fertilizer was applied in fixed pits every year that were dug 30 cm deep on each side of the trunk (1 kg per pit) and then covered with soil. 

### 4.2. Test Materials

The test material was “Zijinhuangcui” (*Prunus persica* cv.). The peach trees were planted in 2017 at a plant spacing of 2 m and a row spacing of 5 m. The live grass mulch comprised ryegrass (*Lolium perenne* L.) and hairy vetch (*Vicia villosa*). A black high-density polyethylene breathable mulching fabric (purchased from Shandong Hennong Plastic Industry Co., Ltd., Dongying, China) was used for the ground fabric mulch.

### 4.3. Test Methods

#### 4.3.1. Soil Management

Grass planting began in the fall of 2016 in a randomized complete block design (RCBD). There were 9 plots of 200 m^2^ each, with 1 row of peach (10 peach trees in each row). Three treatments were designed as follows: black ground fabric mulch (BF), living hairy vetch mulch (HV), and living ryegrass mulch (RG), and they were replicated thrice. The BF mulch covered the sides of the peach tree at a width of 4.5 m on both sides. The first HV mulch was applied at the end of September and the beginning of October 2016. Notably, due to aging and damage to the black ground fabric (the same black ground fabric used initially), the fabric was re-paved in March 2020 and was maintained until the end of the test in 2022. A rotary tiller was used to dig up between and within the rows of the peach trees in the plots before spreading the hairy vetch seeds at a seeding rate of 7.50 g·m^−2^ (5 kg/acre). RG mulch was sowed like hairy vetch and at a similar seeding rate.

The two grass mulches were not mowed during the growing season and were let to grow until they reached a height of 40 cm (cm). The grasses were mowed under the rows only at the beginning of April of the following year using an electric ”Lawnmower” machine. A brush cutter was used to mow the grasses available in the plot every June when the two growing grass species began to disintegrate, followed by covering the soil surface with ryegrass and hairy vetch mulch. At the end of September and beginning of October of the following years, a rotary tiller was used to dig up the soil at the end of September, followed by sowing at the beginning of October of the subsequent years. Figure S3 shows the schematic diagram of the test.

The change in soil physical and biological properties is a slow process. Thus, a long-term experiment (2016–2022) was conducted to investigate this phenomenon. Initially, only basic soil index measurements such as total N, P, K, available N, P, K, pH, and organic matter (Table S1) were performed until significant differences in peach fruits were observed in 2021 (the peach trees started bearing fruit in 2020). Subsequently, in 2022, the experiment was redesigned to take samples at various time points and measure soil indicators and key quality indicators of the fruit. This facilitated the analysis of the relationship between soil properties and peach fruit quality.

#### 4.3.2. Soil Sampling and Collection

Soil samples were collected after the fruit harvest stage (every September from 2017 to 2022) and during the young fruit stage (S1, April 30, corresponding to stage 75 in the BBCH-scale), mature fruit stage (S2, 30 June, corresponding to stage 87 in the BBCH-scale), and after the fruit harvest stage (S3, 30 September) in 2022. We used a drill bit with a length of 20 cm and a diameter of 4 cm to drill holes in the ground during each stage. Five soil cores were randomly collected from at least 10–20 cm between rows in each plot and then mixed into a composite sample. Stones and plant residues were removed from the collected samples. The soil samples were then sieved through a 2 mm sieve and divided into three parts. The first set of subsamples was air-dried and used to determine the soil’s physiochemical properties. The second set of subsamples was preserved in a 4 °C refrigerator and used to determine the available nutrient content, microbial biomass carbon, and nitrogen. The third set of subsamples was preserved in a refrigerator at −80 °C for cryopreservation and used for microbial DNA extraction and community analysis.

#### 4.3.3. Determination of Soil Chemical Properties

Deionized water (0.5–1 mL) and 1.0 g soil was added to the bottom of the digestion tube, then into it we added 2 g accelerator [potassium sulfate (K_2_SO_4_): copper sulfate pentahydrate (CuSO_4_·5H_2_O): selenium powder (Se) = 100:10: 1] and 5 mL sulfuric acid (H_2_SO_4_) for digestion. Subsequently, 20 mL of the above digestion liquid was aliquoted and used to measure the soil total nitrogen (TN) by the Kjeldahl nitrogen analyzer [67]. Next, 5 g soil was weighed and 25 mL potassium chloride (KCl) solution was added to it. The mixture was spun at 180 r/min for 1 h, and then filtered. About 5 mL of soil filtrate was mixed with 2.5 mL of phenol solution and 2.5 mL of sodium hypochlorite (NaClO) solution, then placed at room temperature (20 °C) for 1 h. Finally, 0.5 mL masking agent [mixed 400 g/L potassium sodium tartrate (NaKC_4_H_4_O_6_·4H_2_O) and 100 g/L EDTA disodium salt solution were added in equal volumes, and 0.5 mL of 10 mol/L sodium hydroxide (NaOH) was added to every 100 mL of the mixed solution], then the volume was adjusted to 25 mL with water, and finally, a full wavelength microplate reader (USA MD SpectraMax 190, Shanghai Aiyan Biotechnology Co., Ltd., Shanghai, China) was used to measure the absorbance value at a wavelength of 625 nm to calculate the NH_4_^+^-N content. The soil filtrate was aspirated and then the absorbance values were measured at 220 nm and 275 nm to calculate the NO_3_^−^-N content. To calculate the dissolved total nitrogen (DTN) content, we took 5 mL soil filtrate into a colorimetric tube and added 10 mL alkaline potassium persulfate solution (K_2_S_2_O_8_), then placed the mixture in a high-pressure steam sterilizer, and digested it at 120–124 °C for half an hour. After taking it out, the absorbance was measured at 220 and 275 nm by a full wavelength microplate reader (USA MD SpectraMax 190, Shanghai Aiyan Biotechnology Co., Ltd. Shanghai, China.). To calculate the nitrite content, we measured 3.0 mL soil filtrate and added 0.20 mL chromogenic agent (20 mL sulfonamide solution, 20 mL N(-1-naphthyl)-ethylenediamine hydrochloride solution, and 20 mL concentrated phosphoric acid), and adjusted the volume to 25 mL, then left it to stand for 60–90 min. The absorbance at 543 nm was measured by a full wavelength microplate reader (USA MD SpectraMax 190, Shanghai Aiyan Biotechnology Co., Ltd. Shanghai, China). Dissolved organic nitrogen content (DON) (mg·kg^−1^) was the total dissolved nitrogen (DTN) content minus NH_4_^+^-N, NO_3_^−^-N, and nitrite content. Microbial carbon (MC) and microbial nitrogen (MN) were determined using the chloroform fumigation extraction method [68]. Soil organic matter (SOM) and dissolved organic carbon (DOC) were oxidized using the potassium dichromate + sulfuric acid (K_2_CrO_7_–H_2_SO_4_) method [69]. Under external heating conditions (oil bath temperature is 180 °C, boiling for 5 min), we used 0.4 mol/L K_2_CrO_7_-H_2_SO_4_ solution to oxidize soil organic matter (carbon). The remaining K_2_CrO_7_ was titrated with 0.1 mol/L ferrous sulfate solution (FeSO4·7H_2_O). The soil organic matter (carbon) content was calculated from the amount of K_2_CrO_7_ consumed. Soil total phosphorus (TP) concentration was determined by alkaline digestion (NaOH 2 g, <0.25 mm soil 0.25 g, 400 °C–15 min and 720 °C–15 min) followed by molybdate colorimetric measurement [69]. Total potassium (TK) was measured using a Flame Photometric Detector (FP6450, Shanghai, China) [70]. We weighed 0.2 g of soil, placed it at the bottom of a silver crucible, then added 3–4 drops of absolute ethanol and sprinkled 2 g of sodium hydroxide (NaOH) over the soil. Subsequently, we placed the crucible into a high-temperature electric furnace. When the temperature reached approximately 400 °C, it was paused for 15 min, then the temperature was raised to 720 °C, maintained for 15 min, and then allowed to cool. We then added 10 mL of water at approximately 80 °C. After the frit was dissolved, the solution was transferred carefully to a 50 mL volumetric flask to avoid any damage. Finally, the solution was filtered using phosphorus-free qualitative filter paper. The Flame Photometric Detector (FP6450, Shanghai, China) was used to directly measure the total potassium (TK). In the diffusion dish, 1.2 mol/L NaOH was added to hydrolyze the soil. The soil was hydrolyzed under alkaline conditions and was absorbed by the H_3_BO_3_ solution after diffusion. The NH_3_ in the H_3_BO_3_ absorption solution was then titrated with a 0.01 mol/L hydrochloric acid (HCl) standard solution to calculate the available nitrogen (AN) content. The available phosphorus (AP) in neutral and alkaline soils was extracted using 0.5 M sodium bicarbonate (NaHCO_3_) and analyzed through molybdenum blue colorimetry [71]. Briefly, 2.5 g air-dried soil that passed through a 1 mm sieve was weighed and added into a 100 mL Erlenmeyer flask. We then added 25 mL of 1 mol/L neutral ammonium acetate (NH_4_OAc) solution, shook the mixture for 30 min, and then filtered it with dry ordinary qualitative filter paper. The filtrate was placed in a small Erlenmeyer flask, and the content of available potassium (AK) was measured on a flame photometer. The pH value was measured using a Starter-2100 pH probe (Ohaus, Brooklyn, NY, USA) (the water-to-soil ratio was 2.5:1) [72]. All reagents were purchased from Shanghai McLean Biochemical Technology Co., Ltd. Shanghai, China.

#### 4.3.4. Soil DNA Extraction, Amplification, and Sequencing 

Total deoxyribonucleic acid (DNA) was extracted from 0.5 g of soil using a HiPure soil DNA extraction kit (Magen, Guangzhou, China). Specific primers with a Barcode were subsequently synthesized based on the full-length primer sequence, followed by polymerase chain reaction (PCR) amplification, PCR, product purification, quantification, and normalization to form a sequencing library (SMRT Bell). The constructed libraries were subjected to a library quality check to remove the low-quality libraries, followed by sequencing on a Pacific Biosciences (PacBio Sequel II) platform. The primer sequences used were 341F (5′AGRGTTTTGATYNTGGCTCAG3′) and 806R (5′TASGGHTACCTTGTTASGACTT 3′). The raw read data were further filtered according to the following procedures to obtain high-quality raw read data: (1) Export the PacBio offline data using the Cascading Style Sheets (CCS) file application, perform Barcode recognition on the CCS sequence, filter the length, remove chimeras, and obtain effective CCS; (2) Cluster effective CCS sequences, divide operational taxonomic units/amplicon sequence variants (OTUs/ASVs), hereinafter collectively referred to as features, and obtain species classification based on the sequence composition of features; (3) Perform a taxonomic analysis on the samples at various classification levels based on the feature analysis results and obtain the community structure diagram and species clustering heat map of each sample at the taxonomic level of phylum, class, order, family, genus, and species; (4) Study the species diversity within a single sample through Alpha diversity analysis, and calculate the Chao (community richness) and Shannon (community diversity) index of each sample; (5) Use Beta diversity analysis to compare the species diversity (community composition and structure) differences between different samples. Obtain the samples based on the distance matrix as determined by principal coordinates analysis (PCA and PCoA) diagrams at the corresponding distance; (6) Measure the differences in species abundance composition between different samples (groups) through significance analysis of differences between groups at the species taxonomy composition level; (7) Construct a correlation network and perform redundancy (RDA) analysis based on the composition distribution of species in each sample [73].

#### 4.3.5. Tree Growth Index Measurement

The tree height, crown width, branch length, and trunk thickness of 15 randomly selected trees from each treatment were measured using a benchmark after the defoliation of the peach trees in 2022. These measurements were repeated thrice. The crown width, crown projections, and trunk circumferences above 30 cm of the ground were measured from the east to west poles of the plots using a tape. The branch diameter, internode length, and crown width were measured from the north pole to the south poles of the plot using a vernier caliper.

#### 4.3.6. Determination, Collection, and Sampling of Plant Tissues

A total of sixty mature leaves from the periphery of the crown were randomly selected from each treatment during the young fruit stage (S1, April 30, corresponding to stage 75 in BBCH-scale), fruit mature stage (S2, June 30, corresponding to stage 87 in BBCH-scale), and after product harvest stage (S3, September 30) for laboratory analysis in 2022. The leaves were first washed with clean water, put in paper bags, and dried at 60 °C until they attained a constant weight. The total nitrogen (TN), total phosphorus (TP), and total potassium (TK) contents of the leaves were subsequently measured. About 50 mg of dry leaf fine powder was digested with 5 mL of concentrated sulfuric acid (H_2_SO_4_, high purity grade) in a digestion furnace and ultrapure water was added to the digestion solution to obtain a final volume of 100 mL after digestion. The solution was then filtered using a 0.45 μm sterile needle filter. The TN concentration of the sample was then measured using a Continuous Flow Analyzer (AA3, Bran + Luebbe, Matthews, Vreden, Germany). Approximately 100 mg of dry leaves were ground into fine powder and then mixed with 5 mL of 65% nitric acid (HNO_3_, high purity grade) using a microwave digestion device (MARS 6 CLASSIC, CEM, Matthews, Pittsburgh, PA, USA). Ultrapure water was added to the digestion solution to obtain a final volume of 50 mL after digestion. The solution was then filtered using a 0.45 μm sterile needle filter. The TP and TK concentrations of each sample were then determined using a microwave plasma atomic emission spectrometer (ICP-OES, iCAP™ 7400, Thermo Scientific, Waltham, MA, USA™) [74].

In total, thirty fruits (ten for each treatment) from the periphery of the canopy of each treatment were randomly selected as measurement samples during the mature fruit stage (S2, June 30, corresponding to stage 87 in the BBCH-scale), repeated with ten fruits each. We used a 1/10,000 electronic balance machine to measure the single fruit weight of the peach and a digital vernier caliper to measure the longitudinal, transverse, and lateral diameter of the peach. The content of the fruit color index was measured using a HunterLab ColorQuest XE colorimeter (Hunter Associates Laboratory Inc., Fredericksburg, TX, USA) at four points: the abdomen, back, front, and back side of the fruit. Color brightness was represented by L*, with a value range of 1~100, while color components were represented by a* and b*, with value ranges of −60~60. The a* value denotes a red–green difference index (the positive value represents the degree of red; the greater the positive value, the darker the red, while the negative value represents the degree of green; the smaller the negative value, the darker the green). The b* value denotes a yellow–blue difference indicator (the positive value represents the degree of yellow; the greater the positive value, the darker the yellow, while the negative value represents the degree of blue; the smaller the negative value, the darker the blue). The hue angle (h°) was calculated using the a* and b* values, h° = arctangent b*/a*, (h° is the comprehensive color index, from 0 to 180. It carries different colors, such as purple, red, orange, yellow, yellow-green, green, and blue-green. Among them, h = 0 is purple, h = 90 is yellow, and h = 180 is blue-green). 

Sugar- and acid-related components were determined using an Agilent 1100 high-performance liquid chromatography (HPLC) system (Agilent Technology, Santa Clara, CA, USA) [75]. The sugar component was measured using a CARBOSepCHO-620 CA carbohydrate column (10 µm particle size; 6 × 250 mm; Transgenomic Inc., New York, NY, USA) (column temperature 80 °C) and a refractive index detector (RID). HPLC conditions were as follows: ultrapure water mobile phase, flow rate of 0.5 mL·min^−1^, and injection of 5 µL sucrose, glucose, fructose, and sorbitol were identified and quantified by comparing the retention time and peak area with external standards. The acid-related components were determined using the same HPLC system equipped with a diode array detector and an Agilent ZORBAXSB-Aq column (4.6 × 250 mm ID, 5 µm). Chromatographic analysis was performed at 25 °C with a flow rate of 0.5 mL·min^−1^. The UV absorbance of the eluent was measured at 214 nm. The mobile phase was a 0.02 mol·L^−1^ KH_2_PO_4_ solution with a pH value of 2.7. We injected 5 µL of each sample extract into the HPLC, performed standard identification of malic acid, quinic acid, and citric acid based on retention time, and calculated the organic acid content of the sample based on the standard curve and peak area [76]. The total sugar content was obtained by adding the individual contents of the four types of sugars (sucrose, glucose, fructose, and sorbitol), while the total acid content was obtained by adding the individual contents of the three types of acids (malic, quinic, and citric acids).

### 4.4. Data Analysis

All data were analyzed using the UPARSE software (Uparse v7.0.1001) [77]. The sequences were clustered into operational taxonomic units (OTUs) based on 97% identity, followed by a selection of representative sequences. We calculated alpha diversity metrics, including Chao and Shannon indices, using quantitative insights into microbial ecology (QIIME2). The graphs and maps were created using one-way analysis of variance (ANOVA) in the statistical package for social sciences SPSS 20.0 version software (SPSS Inc., Chicago, IL, USA). The data are presented as means ± standard deviation followed by Tukey’s test (*p* < 0.05) at a confidence level of 95%. At least three independent biological repeats were performed for each measurement. 

## 5. Conclusions

Different mulching treatments had a certain impact on soil nutrients, microbial composition and abundance, tree growth, and fruit quality in peach orchards. Notably, the effects of the living grass mulch treatments (HV and RG), especially the HV treatment, were better than those of the black ground fabric mulch treatment (BF). The living grass mulch treatments increased the contents of TN, AN, MN, SOM, DOC, and MC, significantly improved soil nutrients, and provided a better soil foundation for fruit trees. Among them, the HV treatment had a better comprehensive improvement effect on nitrogen nutrients than the RG treatment. The diversity and abundance of bacteria were also increased under the living grass mulch treatments. HV increased the diversity and abundance of microorganisms more significantly, increasing the abundance of Proteobacteria and *Terrimonas* associated with nitrogen fixation. Microorganisms using nitrogen from the atmosphere promoted the accumulation and circulation of nutrients, thereby enhancing plant height, crown width, continued branch length and tree diameter, accumulation of N, P, and K in the leaves, single fruit weight, and the sugar-acid ratio of the peach fruits. Moreover, the BF and RG treatments promoted fruit coloration, yielding a better effect than that of the HV treatment. The use of living grass mulch treatment is thus an effective strategy for improving soil quality, promoting tree growth, and improving fruit quality in peach orchards.

## Figures and Tables

**Table 1:** The mineral ion content of leaves at different stages of different treatments in 2022.

Physiological Indexes	Period	BF	HV	RG
Total N content of leaves (g·kg^−1^)	S1	28.02 ± 0.37 c	33.09 ± 1.22 a	22.45 ± 0.90 b
	S2	25.64 ± 0.81 b	29.10 ± 0.71 a	25.33 ± 0.68 b
	S3	30.88 ± 1.31 a	30.40 ± 0.54 a	31.20 ± 0.51 a
Total P content of leaves (g·kg^−1^)	S1	2.83 ± 0.20 a	2.71 ± 0.07 a	2.03 ± 0.11 b
	S2	2.26 ± 0.13 b	3.25 ± 0.15 a	3.20 ± 0.13 a
	S3	2.64 ± 0.06 b	2.62 ± 0.20 b	3.58 ± 0.25 a
Total K content of leaves (g·kg^−1^)	S1	19.61 ± 0.13 b	17.82 ± 0.75 c	21.42 ± 0.72 a
	S2	10.82 ± 0.06 b	15.27 ± 0.63 a	14.98 ± 0.80 a
	S3	16.26 ± 0.25 a	17.27 ± 0.73 a	15.89 ± 0.44 a

Note: Values in the table are means ± SD (*n* = 3). BF, black ground fabric mulch; HV, living hairy vetch mulch; RG, living ryegrass mulch. Different lowercase letters indicate significant differences between different mulch treatments in the same period based on the LSD test (*p* < 0.05).

**Table 2:** The external quality of fruit under different treatments at maturity stage.

Treatment	Single Fruit Weight/g	Longitudinal Diameter/cm	Diameter/cm	Side Diameter/cm	L*	a*	b*	h*
BF	132.77 ± 12.98 b	63.36 ± 4.4 b	61.2 ± 5.05 b	61.74 ± 4.78 b	49.4 ± 4.11 a	29.2 ± 0.89 b	26.69 ± 4.35 a	41.53 ± 5.78 a
HV	161.51 ± 19.26 a	66.74 ± 4.85 a	69.82 ± 5.87 a	68.15 ± 4.4 a	46.75 ± 4.98 a	28.67 ± 0.65 b	23.2 ± 5.22 b	37.57 ± 6.3 b
RG	109.85 ± 15.76 c	58.08 ± 3.76 c	55.4 ± 3.86 c	58.36 ± 4.01 c	43.12 ± 3.91 b	31.16 ± 1.27 a	19.68 ± 3.42 c	31.77 ± 2.68 c

Note: Values in the table are means ± SD (*n* = 3). BF, black ground fabric mulch; HV, living hairy vetch mulch; RG, living ryegrass mulch. Different lowercase letters indicate significant differences between different mulch treatments at maturity stage based on the LSD test (*p* < 0.05).

## Data Availability

Data are contained within the article and Supplementary Materials.

## Supplementary Materials

The following supporting information can be downloaded at: https://www.mdpi.com/article/10.3390/plants13060827/s1, Figure S1. Venn diagram of soil bacterial community OTUs under different stages and treatments. Figure S2. Ion content in leaves. Figure S3. Schematic diagram. Table S1. Soil nutrients and physical and chemical properties from 2017–2022. Table. S2. Soil nutrients and physical and chemical properties at different stages of different treatments in 2022. Table S3. The average relative abundances of bacterial phyla at different stages of different treatments in 2022. Table S4. The average relative abundances of bacterial genus at different stages of different treatments in 2022. Table S5. The bacterial diversity index at different stages of different treatments in 2022. Table S6. Growth index of peach tree under different treatments. Table S7. The internal quality of fruit at different stages of different treatments in 2022.
