# Supplementary material for: Effects of Different Mulching Practices on Soil Environment and Fruit Quality in Peach Orchards
Source: Plants (Basel). 2024 Mar 13;13(6):827. doi: 10.3390/plants13060827 (PMC10975533; doi:10.3390/plants13060827)
Supplement: Supplementary file 1 [file plants-13-00827-s001.zip › Peach_grass_Supplementary Figure.pdf]

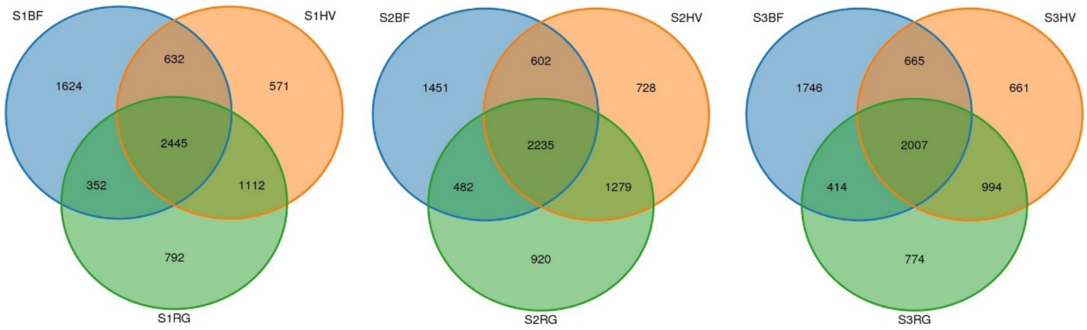

Figure S1. Venn diagram of soil bacterial community OTUs under different stages and treatments. BF: black ground fabric mulch; HV: living hairy vetch mulch treatment; RG: living ryegrass mulch treatment. S1, the young fruit stage (April 30, 2022); S2, fruit mature stage (June 30, 2022); S3, after fruit harvest stage (September 30, 2022); BF, black ground fabric mulch; HV, living hairy vetch mulch; RG, living ryegrass mulch.

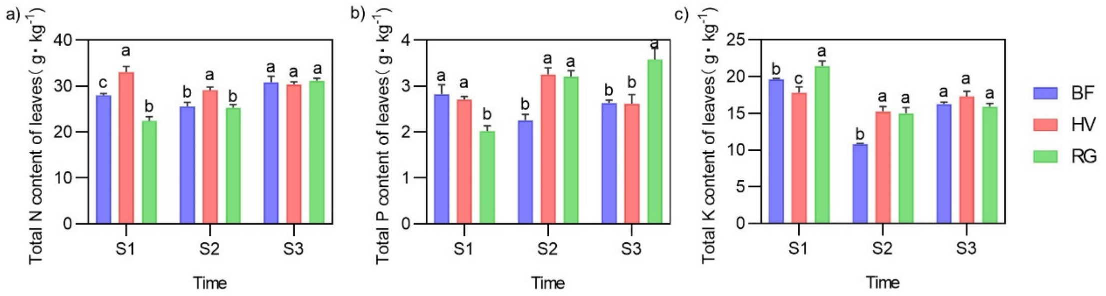

Figure S2. Ion content in leaves. a, total nitrogen content (TN); b, total phosphorus content (TP); c, total potassium content (TK). BF: black ground fabric mulch; HV: living hairy vetch mulch treatment; RG: living ryegrass mulch treatment. S1, the young fruit stage (April 30, 2022); S2, fruit mature stage (June 30, 2022); S3, after fruit harvest stage (September 30, 2022). Boxes with different lowercase letters indicate significant differences between different mulch treatments at the same stage based on the LSD test ($p < 0.05$).

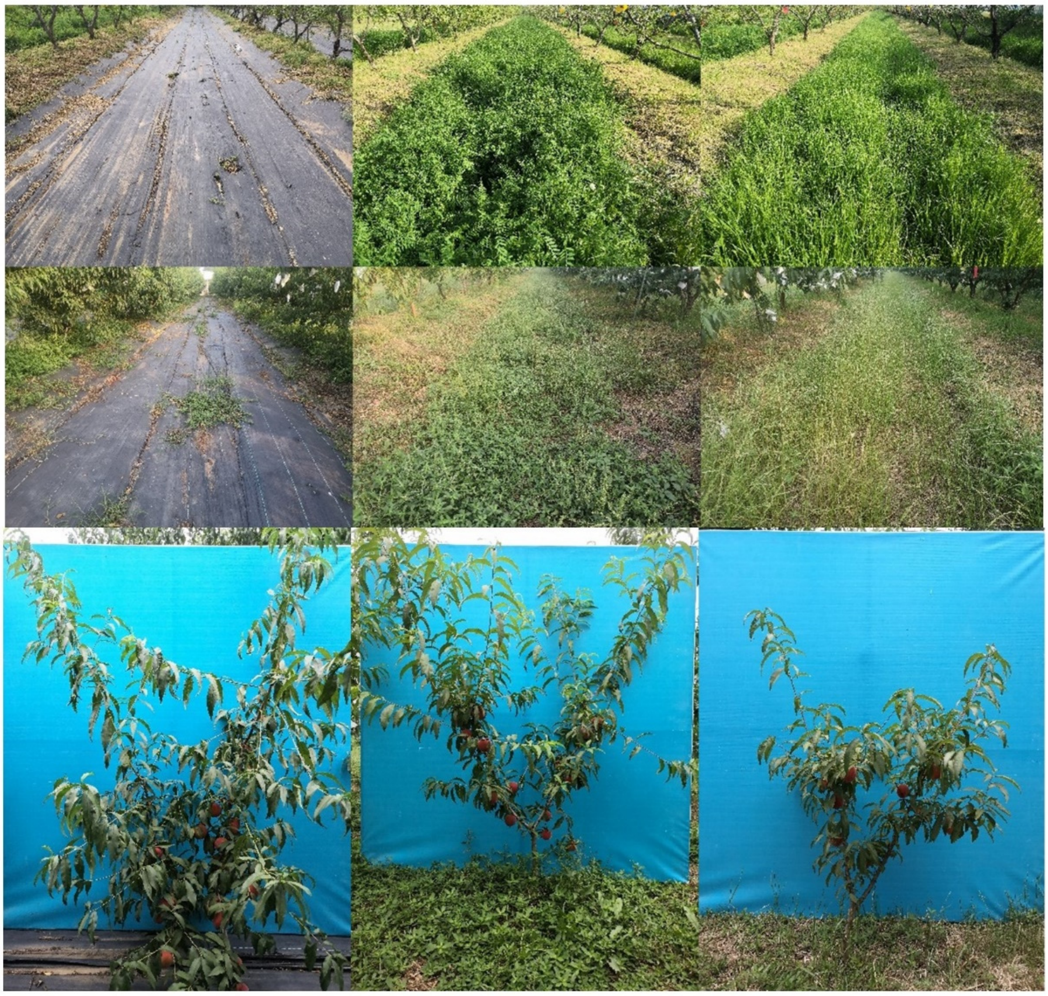

Figure S3. Schematic diagram. BF: black ground fabric mulch; HV: living hairy vetch mulch treatment; RG: living ryegrass mulch treatment.
